# Sensitive Morphological Characterization of Oriented High‐Density Lipoprotein Nanoparticles Using ^31^P NMR Spectroscopy

**DOI:** 10.1002/anie.202004130

**Published:** 2020-08-17

**Authors:** Sophie Lau, David A. Middleton

**Affiliations:** ^1^ Department of Chemistry Lancaster University Lancaster LA1 4YB UK

**Keywords:** apolipoprotein A-I, circular dichroism, dynamic light scattering, lipids, nanoparticles, NMR spectroscopy

## Abstract

The biological function of high‐density lipoprotein (HDL) nanoparticles, the so‐called good cholesterol that is associated with a low risk of heart disease, depends on their composition, morphology, and size. The morphology of HDL particles composed of apolipoproteins, lipids and cholesterol is routinely visualised by transmission electron microscopy (TEM), but higher‐resolution tools are needed to observe more subtle structural differences between particles of different composition. Here, reconstituted HDL formulations are oriented on glass substrates and solid‐state ^31^P NMR spectroscopy is shown to be highly sensitive to the surface curvature of the lipid headgroups. The spectra report potentially functionally important differences in the morphology of different HDL preparations that are not detected by TEM. This method provides new morphological insights into HDL comprising a naturally occurring apolipoprotein A‐I mutant, which may be linked to its atheroprotective properties, and holds promise as a future research tool in the clinical analysis of plasma HDL.

## Graphical abstract

^31^P NMR spectra are highly sensitive to the morphology of high‐density lipoprotein nanoparticles when oriented on glass surfaces. The spectra reveal new molecular insights that are relevant to the protective function of the lipoprotein nanoparticles against cardiovascular disease.

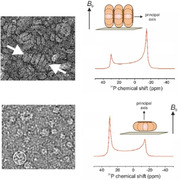

“Good cholesterol” is the familiar term for high‐density lipoproteins (HDL), the heterogeneous proteolipid nanoparticles that transport cholesterol from peripheral tissue to the liver for excretion in a process called reverse cholesterol transport (RCT).^1^ This popular name reflects the long‐held belief that high blood levels of HDL cholesterol (HDL‐C) correlate with a low risk of developing cardiovascular disease, but this notion is now being challenged. Individuals with very high HDL‐C may be at increased risk of developing cardiovascular disease,^2^ and therapies to raise circulating HDL‐C have not shown significant improvements in cardiovascular health.^3^ Additionally, individuals carrying certain mutations of the main HDL protein, apolipoprotein A‐I (apoA‐I), including the Milano (R173C)^4^ and Zaragoza (L144R)^5^ variants, have a low predisposition to cardiovascular disease, despite having naturally low levels of circulating HDL‐C. There is increasing evidence that the *quality* of HDL‐ broadly defined by size, composition and morphology^6^—is a better predictor of cardiovascular health than is the *quantity* of blood HDL.^7^ HDL exists as nascent discoidal or mature spherical assemblies of bilayer phospholipids, triglycerides and cholesterol/cholesteryl esters surrounded by a belt of α‐helical apoA‐I.^8^ The transient morphology of HDL influences their lipid‐binding properties^9^ and metabolism,^10^ and the surface curvature of the lipid cargo regulates interactions with cholesterol‐handling enzymes.^11^ HDL morphology is routinely visualised by TEM, but higher‐resolution techniques sensitive to subtle morphological transformations would offer new, valuable mechanistic, diagnostic and therapeutic insights.

Here we demonstrate an oriented‐sample solid‐state ^31^P NMR method that reports on HDL morphology by virtue of its sensitivity to the surface curvature of the phospholipid headgroups. We characterise reconstituted HDL (rHDL) formulations of defined composition mimicking the nascent form of plasma HDL and comprising a belt of 2 apoA‐I molecules and palmitoyloleoylphoshatidylcholine (POPC) lipid cargo. High‐resolution solution NMR restraints have previously been used to construct structural models of rHDL‐like (membrane scaffold protein) nanodiscs, formed with shorter apoA‐I variants to increase reorientation rates,^12^ but the gain in resolution comes at the expense of averaging the ^31^P chemical shift anisotropy (CSA) information that reports the lipid headgroup curvature. The CSA is preserved in the case of bicelles or larger diameter macrodiscs when magnetically aligned in solution,^13^ but this approach is not suitable for HDL particles, which are smaller and do not align in a magnetic field. We show here that rHDL particles adopt specific orientations on planar glass surfaces, and that the specific orientations of the lipid headgroup in the applied magnetic field, *B*_0_, yield NMR line shapes that are sensitive to the surface curvature of the lipids. Oriented planar lipid membranes have been exploited widely in NMR structural studies of membrane‐embedded proteins.^14^ The new application of this approach to the analysis of HDL morphology exploits the relationship between the mean, dynamically‐averaged lipid orientation relative to *B*_0_ (defined by angle β) and the ^31^P NMR lineshape.

Visualisation of an rHDL preparation of 200:2 POPC:apoA‐I WT by uranyl acetate‐stained TEM reveals that the particles form rouleaux, or regular arrays of ovoid disc‐like structures with the disc face perpendicular to the grid surface (Figure 1 A). These stacked structures are often observed in phosphotungstate‐stained TEM images of lipoproteins and can be eliminated using optimised protocols employing negative staining with uranyl salts.^15^ Similar rouleaux are seen in images of POPC:cholesterol:apoA‐I WT and POPC:apoA‐I L144R (Figure 1 A). When the 200:2 POPC:apoA‐I WT preparation is deposited on TEM grids at a lower concentration the rouleaux are eliminated and only discrete, annular particles are observed with the face of the discs lying along the grid surface (Figure 1 A, right hand image). Hence, with uranyl acetate staining, rouleau formation appears to be predominantly a function of HDL concentration. We reasoned that the rHDL nanodiscs deposited on planar glass surfaces adopt the same concentration‐dependent orientations as on the TEM substrate.

**Figure 1:**
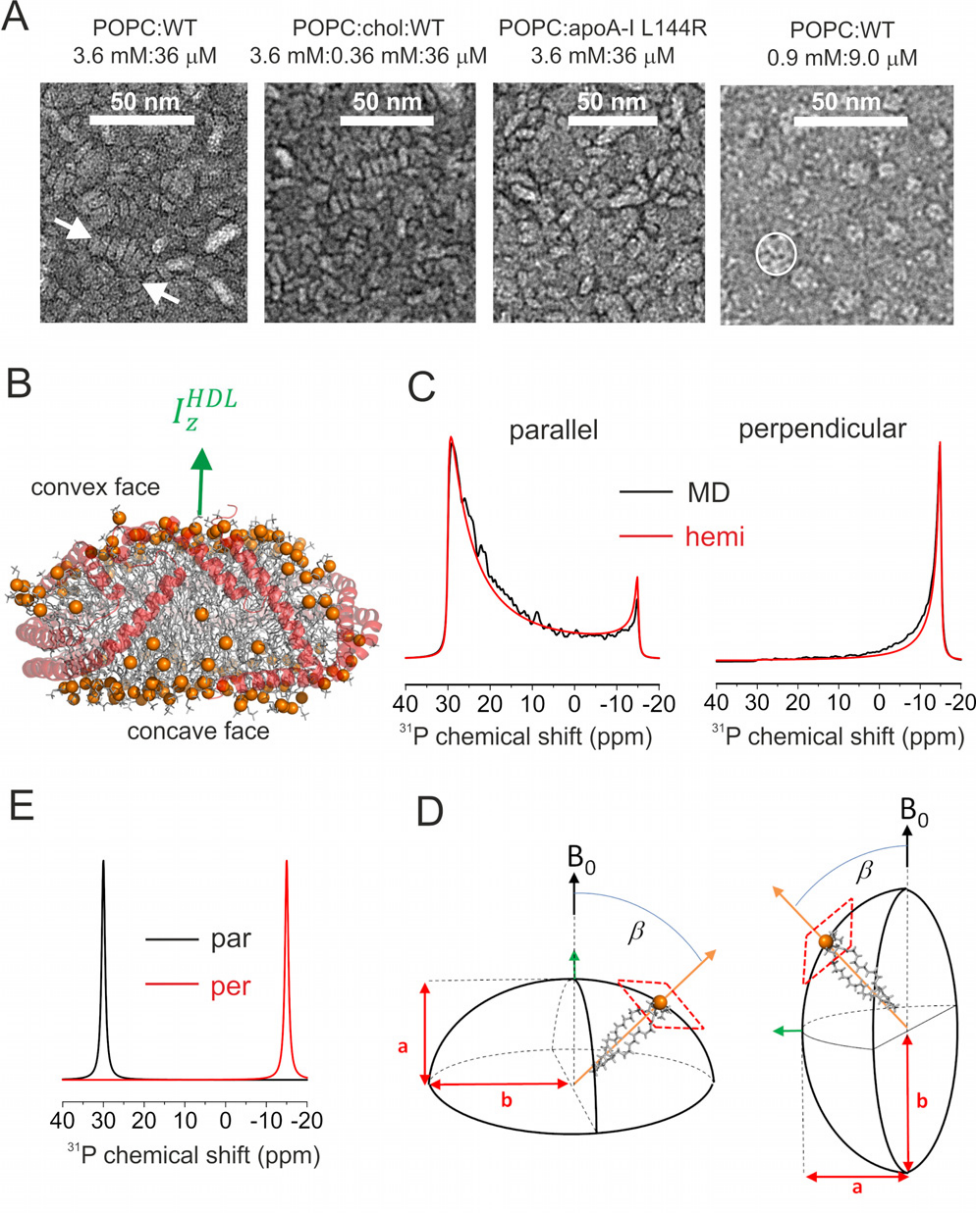
The morphology of rHDL nanoparticles and its relationship to ^31^P NMR line shape. A) Negative‐stain TEM images of different rHDL formulations. B) Final frame after 10 μs MD simulation of a 200:20:2 POPC:cholesterol:apoA‐I assembly.^16^ Orange spheres denote the lipid phosphate groups, the lipid acyl chains are grey, and the apoA‐I molecules are red. C) Simulated line shapes for the HDL principal axis of inertia $I_{z}^{HDL}$ aligned parallel with or perpendicular to *B*_0_, based on the MD model (black) or hemispheroid approximation (red). D) The corresponding oblate hemispheroid (*a*/*b*=0.4) representing the lipid orientational distribution, with minor axis *a* parallel with or perpendicular to *B*_0_. E) Simulated lineshapes for planar lipid bilayers with bilayer normal parallel with or perpendicular to *B*_0_. A line broadening of 100 Hz was applied to all simulations.

To establish an initial relationship between disc orientation, morphology and ^31^P NMR line shape, we analysed lipid orientations in models of a 200:20:2 POPC:cholesterol:apoA‐I assembly obtained with atomistic molecular dynamics (MD) simulations over a 10 μs trajectory^16^ (Figure 1 B). In the MD models, the double belt of helical apoA‐I adopts a zig‐zag arrangement to accommodate the lipid‐protein circumference mismatch,^16^ which bends the lipid bilayer such that one surface is concave and the other surface convex (Figure 1 B) to maintain contact between the two leaflets. ^31^P NMR line shapes were calculated from the average lipid orientations over the final 1 μs of the MD trajectory (Figure 1 C), with the HDL principal axis of inertia $I_{z}^{HDL}$ oriented parallel with *B*_0_ or perpendicular to *B*_0_ (as in rouleaux, with the edges of the stacked particles contacting the surface). Analysis of the MD models reveals that lipids closest to the disc centre are oriented with their long molecular axes approximately parallel with $I_{z}^{HDL}$ , whereas the outer lipids favour perpendicular orientations with respect to $I_{z}^{HDL}$ (Figure S1). We considered whether the lipid distribution of the MD model may be approximated by an oblate hemispheroid in which the average lipid molecular orientation at any point on the surface is given by the normal to the tangent plane at that point (Figure 1 D).^17^ Simulated lineshapes based on this approximation (Figure 1 C) are in good agreement with the lineshapes calculated from the MD models, and in both cases the lineshape asymmetry reflects the HDL surface curvature. As the curvature decreases (defined by an increase in flattening factor *f*=1−*a*/*b* from zero toward 1.0), the lineshapes become less asymmetric (Figure S2) until fully planar bilayers may be represented by Lorentzian lines at the two extremes of the ^31^P CSA (Figure 1 E). rHDL comprising 200:2 POPC:apoA‐I WT (comparable to the TEM preparation) was dried on glass microscope cover slips and rehydrated in a 99 % humidity environment. The ^31^P NMR spectra of stacked slides held at 90° to *B*_0_ have Lorentzian lineshapes, consistent with the rHDL lipids in essentially planar bilayers (i.e., *f*=1.0) rather than the surface curvature predicted by MD. The lipids must therefore be sufficiently relaxed within the interior to avoid distorting from planarity. The presence of two peaks indicates that populations of rHDL particles exist in parallel and perpendicular orientations (Figure 2 A). After longer rehydration times, the lineshapes remain Lorentzian but become narrower, suggesting a reduction in the lipid orientational distribution, and the peak at 30 ppm becomes increasingly dominant. The rehydration time does not alter the planar morphology of the particles, but longer rehydration times promote their reorientation, possibly by disassembly of rouleaux into discrete particles (Figure 2 A). After 16 h rehydration the separation of the two peaks decreases by ≈4 ppm. This is probably due to a reduction in the CSA because of increased lipid mobility at the higher hydration level.

**Figure 2:**
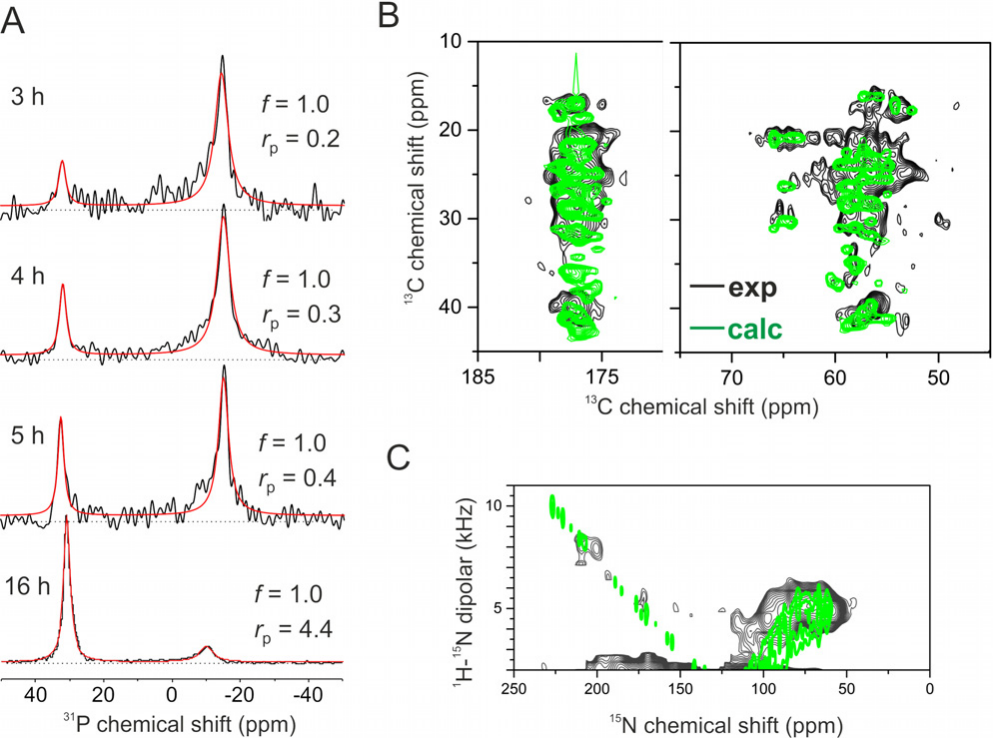
NMR spectra of rHDL particles (200:2 POPC:apoA‐I WT, 3 mm lipid). A) ^31^P NMR spectra of oriented rHDL samples rehydrated for 3–16 h. The best fitting Lorentzian lineshapes (red) were obtained by optimising the linewidths and peak areas. The areas of the two peaks are proportional to the variable *r*_p_ representing the ratio of rHDL particles oriented with the bilayer normal parallel with *B*_0_ to particles oriented perpendicular to *B*_0_. B) Regions of a high‐resolution 2D ^13^C‐^13^C NMR spectrum of PEG‐precipitated rHDL (containing [U‐^13^C]apoA‐I) obtained with 8 kHz magic‐angle spinning (MAS) and 100 ms DARR mixing. Overlaid are calculated cross‐peaks (green) at the chemical shifts predicted from the model in Figure 1 B. C) A ^1^H‐^15^N PISEMA spectrum of oriented rHDL particles (containing [U‐^15^N]apoA‐I) after rehydration for 16 h. The overlaid simulated spectrum (green) is based on the ^15^N chemical shifts and ^1^H‐^15^N dipolar couplings calculated from the model in Figure 1 B with $I_{z}^{HDL}$ parallel with *B*_0_. See Supporting Information for details of all calculations.

We next analysed the orientation of the apoA‐I protein belt relative to the lipid bilayer in the 200:2 POPC:apoA‐I WT preparation. In early models of discoidal HDL, the apoA‐I helices are approximately parallel with the lipid bilayer normal (e.g., ref. 18) but later MD‐ and experimentally‐ derived models^12^, ^16^ support the belt arrangement in Figure 1 B. A magic‐angle spinning (MAS) ^13^C‐^13^C dipolar correlation NMR spectrum of non‐oriented, precipitated rHDL containing [U‐^13^C]apoA‐I (Figures 2 B and S3) agrees with a spectrum calculated from the structural model in Figure 1 B and reflects the largely helical protein structure.^16^ Taking advantage of 16 h rehydration to attain the parallel orientation of rHDL relative to *B*_0_ (Figure 2 A, bottom), we used ^1^H‐^15^N PISEMA NMR to determine the orientation of the [U‐^15^N]apoA‐I helices relative to the lipids (Figure 2 C). The experimental spectrum is consistent with the structure in Figure 1 B oriented with the nanodisc principal axis parallel to *B*_0_, as seen by comparison with a calculated spectrum.

Next we asked whether the apoA‐I mutants, R173C and L144R, alter the lipid surface curvature of rHDL, providing a physical basis for their atheroprotective properties. We compared ^31^P NMR spectra of rHDL comprising 200:2 POPC:apoA‐I WT (rHDL WT), L144R (rHDL L144R) and R173C (rHDL R173C). The particles were deposited at a lower concentration to attempt to minimise rouleau formation and triplicate samples (#1–#3) were analysed to confirm reproducibility (Figure 3 A). The spectra of rHDL WT again have Lorentzian lineshapes expected for planar bilayers. The ratio, *r*_p_, of the two peak areas varies in each spectrum, which reflects differences in the proportions of particles in the parallel and perpendicular orientations. The spectra of rHDL‐R173C also have Lorentzian lineshapes, indicating that the particles are morphologically similar to rHDL WT. The ratio *r*_p_ is lower than for rHDL WT, however, suggesting that the particles have a higher propensity to form rouleaux. A strikingly different, broad asymmetric lineshape is seen for rHDL L144R. The 30 ppm edge extends toward the −15 ppm extreme, which is reminiscent of the lipid curvature in the MD model (Figure 1 B) and the lineshape is consistent with a hemispheroid of *f*=0.5–0.6. The lower *r*_p_ values for rHDL L144R indicate a lower propensity of this form to adopt rouleaux.

**Figure 3:**
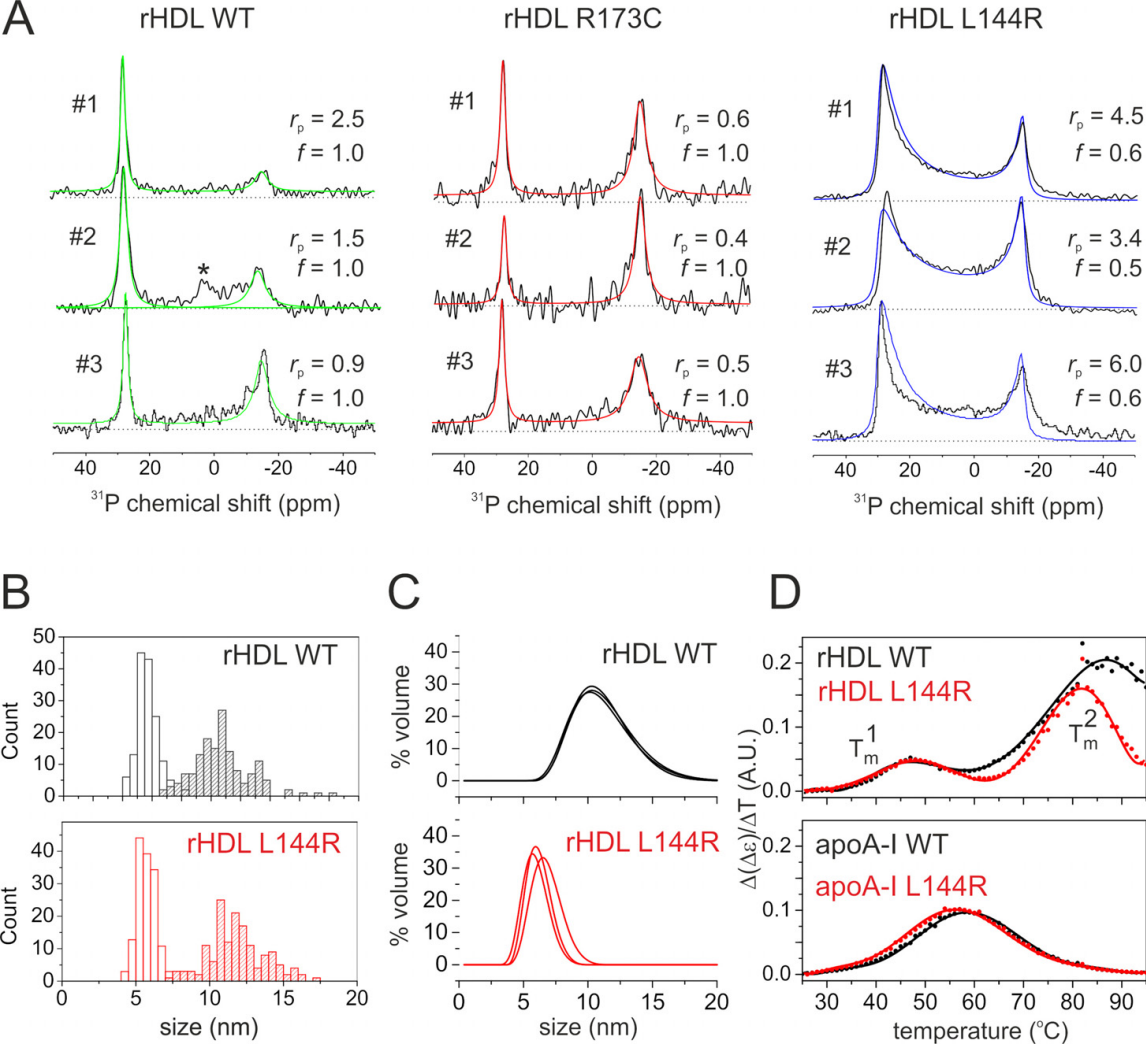
Comparison of rHDL comprising WT and mutant apoA‐I. A) ^31^P NMR spectra of oriented rHDL after rehydration for 4 h. Spectra (black) are overlaid with the best‐fitting simulated line shapes (green and red: Lorentzian; blue: hemispheroid) obtained with *f*, *r*_p_ and line broadening as the fitting parameters, except for rHDL L144R, where a broadening of 200 Hz was applied. Each spectrum, #1–#3, is from a different sample. The asterisk denotes a peak from additional component not seen elsewhere. B) Distribution of rHDL widths (open columns) and diameters (shaded columns) measured from TEM images. C) Volume‐weighted size distributions (in triplicate) calculated from dynamic light scattering (DLS) diffusivity measurements. D) Thermal stability of rHDL and lipid‐free apoA‐I measured by circular dichroism (CD) spectroscopy.

We investigated possible reasons for the different morphologies of rHDL WT and rHDL L144R, by examining apoA‐I structure and rHDL particle size and stability. High‐resolution 2D ^13^C‐^13^C MAS NMR spectra of precipitated rHDL‐WT and L144R are very similar (Figure S4), suggesting only subtle structural differences between the two protein forms. According to CD measurements, apoA‐I in rHDL‐L144R has a 12 % lower α‐helical and 10 % more unordered content than in rHDL WT (Table S1). Native electrophoretic gels exhibit diffuse bands from which particle diameters of 9.0–11.0 nm for rHDL L144R and 9.4–10.0 nm for rHDL‐WT were estimated (Tables S2 and S3, Figure S5), whereas TEM measurements (Figure 3 B) yield mean diameters of ≈11.0 nm for both particles. However, as the mean thickness measured by TEM (≈5.5 nm) is greater than expected for a hydrated lipid bilayer (≈4.1 nm), the dimensions may be overestimated. DLS diffusivity measurements (Figure 3 C) are consistent with a large apparent difference in the mean hydrodynamic diameters, $\bar{d}$_H_, of rHDL‐L144R ($\bar{d}$_H_=6.5 Å) and rHDL‐WT ($\bar{d}$_H_=10.5 Å). That such a difference is reported by DLS and not by TEM may be due to the particles having different size distributions when hydrated and when dried for TEM. However, the shape‐dependent diffusion properties of the planar discoidal rHDL WT and the curved rHDL L144 may also play a role. The standard calculation of *d*_H_ using the Stokes‐Einstein relation can overestimate the diameters of non‐spherical particles.^19^ Modified equations that better describe the diffusion of ellipsoidal and discoidal particles^19^ suggest that the rHDL WT disc diameter may be overestimated considerably when using the spherical approximation, whereas the measured diameter of rHDL L144R may be closer to the actual diameter (see Supporting Information). Finally, variable‐temperature CD reveals two thermal transitions at temperatures $T_{m}^{1}\;$ and $T_{m}^{2}$ (Table S4, Figures S6 and S7), which have been attributed to secondary structural rearrangements of apoA‐I, followed by protein‐protein or protein‐lipid dissociation.^20^ The lower $T_{m}^{2}\;$ for rHDL L144R suggests that the out‐of‐plane distortion of the rHDL L144R lipids reduces the overall rHDL stability, which may have implications for the efficiency of lipid transfer in vivo.

We next examined whether the ^31^P NMR lineshape is sensitive to rHDL containing cholesterol at different concentrations. The ^31^P NMR spectra of rHDL containing 10:1 POPC:cholesterol (Figure 4 A) have two outer components indicating populations of particles in two orientations. As the lipid:cholesterol:protein ratio increases from 160:16:2 to 300:30:2, the peak at −15 ppm curves progressively toward the 30 ppm limit, reflecting increasing deformation from a planar lipid distribution. The lineshapes do not conform to the hemispheroid distribution in which the average tilt angle of each lipid depends on its position from the centre. The lineshape is better described by a discoidal model in which a planar bilayer core is surrounded by lipids in a torus distribution. The outer lipids are forced further away from the bilayer normal as the number of lipids increases (Figures 4 A and S8). A spectrum of rHDL at a high lipid:protein ratio (300:2) in the absence of cholesterol also conforms to this model (Figure 4 B), This alternative morphology may accommodate molecular crowding by increasing the lipid surface area and minimise aqueous exposure of the boundary lipid chains.

**Figure 4:**
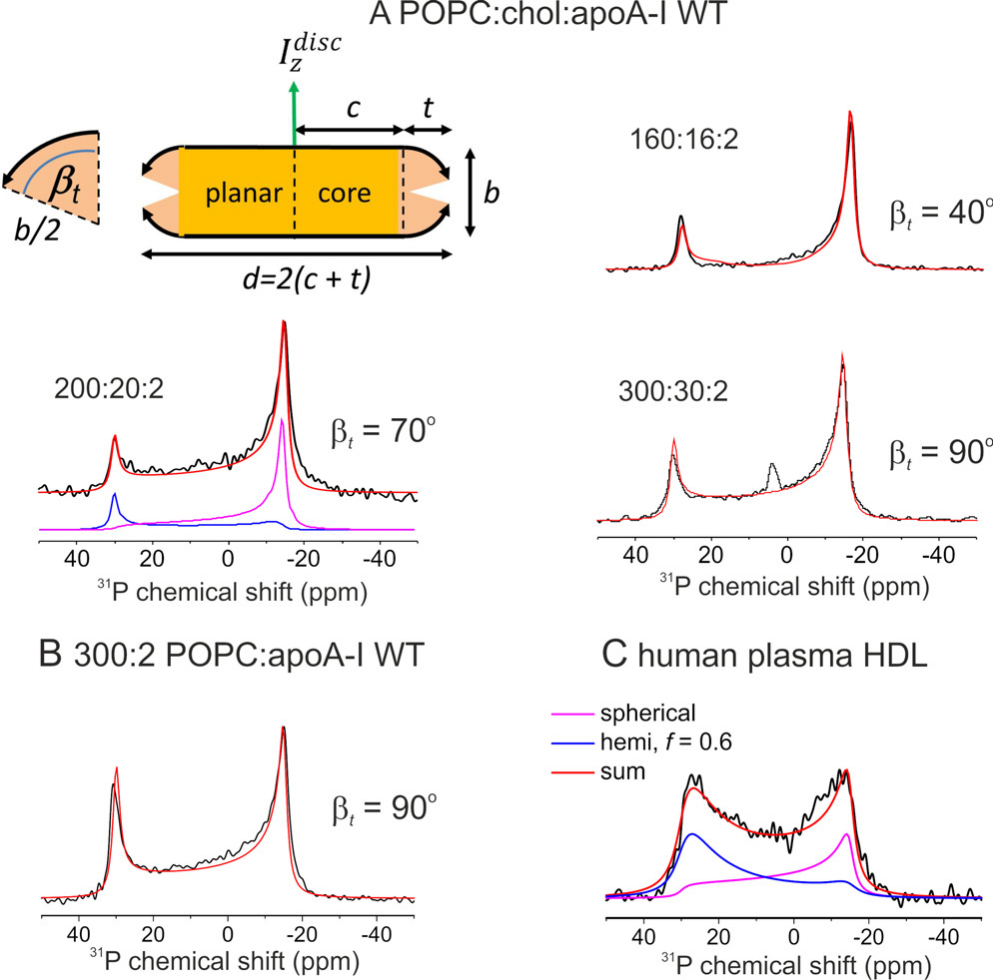
The effect of lipid and cholesterol composition on the ^31^P NMR spectra of oriented HDL particles. A) Spectra of rHDL of different POPC:cholesterol:apoA‐I ratios (3 mm lipid) and the best fitting simulated spectra (red). Lineshapes were calculated for the discoidal model as described in the main text, by summation of the components representing $I_{z}^{disc}$ oriented parallel with and perpendicular to *B*_0_ (e.g., as shown by the blue and purple lines, respectively). B) Spectrum of 300:2 POPC:apoA‐I and best fitting calculated lineshape. C) Spectrum of unfractionated human plasma HDL. The red line is the sum of a lineshape for a hemispheroid and a component for a fully spherical distribution in equal proportion. Samples were rehydrated for 4 h before the NMR measurements.

In summary, oriented sample ^31^P NMR is highly sensitive to the shape of rHDL, and has distinguished three distinct morphological species associated with different HDL composition that are not evident in TEM images. This method will add an extra dimension to structure‐function studies of HDL and its interactions with lipids, cholesterol and RCT enzymes. Further, a spectrum of unfractionated plasma HDL (Figure 4 C) reveals distinctive features reflecting the distribution of mature spherical and flatter, nascent particles. With more rigorous sample preparation and size fractionation, this method promises to be of diagnostic value in cardiovascular disease, by reporting the morphological characteristics of HDL from human patients.

## Conflict of interest

The authors declare no conflict of interest.

## Supporting information

As a service to our authors and readers, this journal provides supporting information supplied by the authors. Such materials are peer reviewed and may be re‐organized for online delivery, but are not copy‐edited or typeset. Technical support issues arising from supporting information (other than missing files) should be addressed to the authors.

Supplementary
