# Supplementary material for: Sensitive Morphological Characterization of Oriented High‐Density Lipoprotein Nanoparticles Using 31P NMR Spectroscopy
Source: Angew Chem Int Ed Engl. 2020 Aug 17;59(41):18126–30. doi: 10.1002/anie.202004130 (PMC7589421; doi:10.1002/anie.202004130)
Supplement: Supplementary file 1 — Supplementary [file ANIE-59-18126-s001.pdf]

Angewandte Chemie
Eine Zeitschrift der Gesellschaft Deutscher Chemiker

Supporting Information

Sensitive Morphological Characterization of Oriented High-Density Lipoprotein Nanoparticles Using ^{31}P NMR Spectroscopy

*Sophie Lau and David A. Middleton**

anie_202004130_sm_miscellaneous_information.pdf

Contents

1. Methods and Materials

1.1 Protein production

1.2. Production of rHDL particles

1.3. Density ultracentrifugation of rHDL

1.4. Analysis of rHDL size by gel electrophoresis

1.5. Transmission electron microscopy

1.6. Preparation of oriented rHDL

1.7. PEG Precipitation of rHDL

1.8. Circular dichroism

1.9 Dynamic light scattering

1.10. Solid-state NMR

1.11. Calculations of ^{31}P NMR lineshapes

1.11.1. Planar and spherical lipid distributions

1.11.2. Lineshapes calculated from MD models

1.11.3. The hemispheroid approximation

1.11.4. Simulations of discoidal particles.

1.11 Calculations of two dimensional NMR spectra

2. Tables

3. Supplementary figures

4. Supplementary references

1. Methods and Materials

1.1 Protein production

Expression of N-terminally His-tagged apoA-I was carried out by following previously published methods.[1] A pNFXex expression vector coding for human apoA-I with an N-terminal His tag (kindly provided by Dr M. Oda, Oakland Research Institute, USA) was transformed into E. *coli* BL21 (DE3) cells (Agilent Technologies). The plasmid construct expresses apoA-I with an E2D mutation enabling removal of the His-tag by cleavage of the acid-labile Asp2-Pro3 peptide bond with formic acid, leaving the native residues 3-243. The apoA-I R173C and L144R mutants were produced using SLIM PCR[2], the sequence and mass were confirmed via gene sequencing and mass spectrometry, respectively. The apoA-I mutants were expressed following the same procedure as apoA-I WT.

The cells were grown at 37°C at 200 rpm in LB media containing 100 µg/ml ampicillin (Melford Laboratories Ltd) until the OD_{600} ≥ 0.6. Protein expression was induced with the addition of 1 M IPTG (Melford Biolaboratories Ltd) to a final concentration of 1 mM, and incubated at 37°C, at 200 rpm for 5 hours. The cells were centrifuged at 5000 x g for 20 minutes. To lyse the cells, the pellet was resuspended in lysis buffer (6 M guanidine HCl, 20 mM $NaPO_4$, 0.5 M NaCl) pH 7.4, and frozen at -20°C overnight. Before sonication of the cell lysate PMSF (200 mM stock) was added to a final concentration of 1 mM. To further lyse the cells, the sample was sonicated for 5 cycles on ice, with intervals of 15 seconds on and 60 seconds off at an amplitude of 22 microns. The cell debris was removed by centrifugation at 43 000 x g for 30 minutes at 4°C.

His-trap chromatography using an ÄKTA Start chromatography system and UNICORN Start 1.0 software (GE Healthcare) was used to purify the fusion protein. The 5 ml HisTrap FF pre-packed nickel Sepharose column (GE Healthcare) was washed with dH_2O and equilibrated with lysis buffer pH 7.4, at a flow rate of 5 ml/min. The lysate was diluted with an equal volume of lysis buffer pH 7.4 before loading onto the column at the flow rate of 5 ml/min, and 10 ml fractions were collected. The column was washed in succession with lysis buffer pH 7.4, and binding buffer pH 7.4 (20 mM $NaPO_4$ and 0.5 M NaCl) containing an increasing concentration of imidazole to wash non-specific binding proteins (20 mM and 45 mM

imidazole) and elute the apoA-I fusion protein (500 mM imidazole). Fractions containing the fusion protein were dialysed (12 - 14 kDa MWCO tubing) overnight in dialysis buffer pH 7.4 (50 mM Tris-HCl, 1 mM EDTA, 1 mM benzamidine hydrochloride) at 4°C.

Formic acid 45% (v/v) was added to the fusion protein and the solution was heated at 55°C for 5 h. To separate the cleaved his-tag and the apoA-I protein, the solution was dialysed in 5 L of dialysis buffer pH 8.0 overnight twice, at 4°C with 1 buffer change. Additionally for apoA-I R173C only, the protein was incubated with 5 mM TCEP (Sigma-Aldrich) for 24 hours at 4°C to remove glutathione, a post-translational cysteine modification[3], and dialysed with at least 2 buffer changes to remove the TCEP and glutathione. After dialysis the protein was incubated for 48 hours at 4°C to allow dimer formation. The concentration of apoA-I was measured using the NanoDrop™ 2000 spectrophotometer (Thermo Scientific) and stored at -20°C until use.

For the expression of uniformly ^{13}C and/or ^{15}N labelled apoA-I the cells were grown in LB media containing 100 ug/ml ampicillin until the OD_{600} ≥ 0.6 and pelleted by centrifugation at 5000 x g for 20 minutes. The cells were resuspended in 1 L of pre-warmed minimal media, pH 7.2 (22 mM D-Glucose-$^{13}C_6$ (Sigma-Aldrich), 19 mM ^{15}N-ammonium chloride (CortecNet), 0.3 mM ampicillin, 0.1 mM $CaCl_2$, 0.03 mM thiamine hydrochloride, 1 mM $MgSO_4$, 88 mM Na_2HPO_4, 55 mM KH_2PO_4). After a 30-minute equilibration at 37°C at 200 rpm the same procedure was followed for inducing protein expression and purification as described for unlabelled apoA-I.

1.2. Production of rHDL particles

The widely established detergent mediated sodium cholate dialysis method was used in the production of rHDL particles.[4] POPC (Avanti®) and cholesterol (if used) were dissolved in chloroform to a final molar ratio of POPC/Cholesterol 10:1 (M/M) and dried with N_2 to form a thin film. To remove any residual chloroform the sample was placed under a high vacuum for a minimum of four hours.

All samples were prepared in rHDL buffer pH 7.4 (10mM Tris base, 1mM EDTA, 1mM NaN_3). The lipid only or lipid/cholesterol films were resuspended with rHDL buffer containing sodium cholate (30 mg/ml stock) to a final lipid: detergent molar ratio of 1:1 (M/M). The solution was incubated for 2 hours at 4°C with stirring

until clear. ApoA-I was added to the POPC/detergent mixture at a protein: lipid molar ratio of 1:100 (M/M) and incubated overnight at 4°C. Samples were transferred to dialysis tubing (12 - 14 kDa MWCO) and dialysed against buffer to remove the sodium cholate, with a total of 5 buffer changes at a 500-fold excess volume over at least 3 days.

The POPC lipid concentration at each stage of the sample preparation was measured in duplicate using the phospholipid quantification assay kit (CS0001, Sigma-Aldrich®). The cholesterol concentration was measured in duplicate using the cholesterol quantitation kit (MAK043, Sigma-Aldrich®). The protein concentration was measured in triplicate using the NanoDrop™ 2000 spectrophotometer (ThermoFisher Scientific).

1.3. Density ultracentrifugation of rHDL

Sodium bromide flotation or discontinuous gradient density ultracentrifugation was used to separate lipid-free apoA-I and the rHDL particles. The methods were adapted from protocols used to separate plasma lipoproteins and heterogenous rHDL particles.[4-5]

In flotation ultracentrifugation the rHDL sample density was increased to greater than 1.21 g/ml with the addition of 1.5 ml of 38 % (w/v) NaBr to 0.5 ml of rHDL particles. In a 10.4 ml centrifuge tube (Beckman Coulter), the solutions were under-layered in the tube (top to bottom), 0.5 ml of rHDL buffer, 7.9 ml of 1.21 g/ml density NaBr solution, and 2.0 ml of sample. The samples were centrifuged at 65,000 rpm for 22 hours at 4°C in a type 70.1 Ti rotor (Beckman Coulter). After centrifugation five 0.5 ml aliquots were collected from the top of the solution. The fractions were dialysed at 4°C to remove the NaBr with at least two buffer changes over 2 days.

In step gradient density ultracentrifugation, sodium bromide solutions were under-layered in the following order 1.04, 1.09, 1.15, 1.21 g/ml densities. The final layer consisted of 0.5 ml of rHDL sample and 1.5 ml of 38 % (w/v) NaBr to increase the sample density to greater than 1.21 g/ml. The samples were centrifuged at 41, 000 rpm for 24 hours at 4°C in a SW41 Ti rotor (Beckman Coulter). After centrifugation each NaBr density layer was pooled (~2.5 ml) and dialysed against rHDL buffer, pH 7.4 to remove the NaBr.

The presence of rHDL particles in the pooled fractions was confirmed by native gradient gel electrophoresis. The sample concentrations were increased using an Amicon Ultra-4 device with a 10,000 kDa MWCO (Merck). The spin concentrator was rinsed with dH_2O before the sample was centrifuged at 4000 x g for 30 minutes to reduce the sample volume for rHDL PEG precipitation.

1.4. Analysis of rHDL size by gel electrophoresis

Non-denaturing gradient gel electrophoresis (NDGGE) was used to determine the homogeneity and size of the rHDL nanoparticles formed. The NDDGE gels were 4-16% NativePAGE Bis-Tris protein gels (Invitrogen) and the marker was the HMW calibration kit for native electrophoresis (Amersham, GE Healthcare).[5b, 6] The marker lane contains the HMW marker kit (GE Healthcare) proteins with the corresponding Stokes' diameters thyroglobulin (17.0 nm), ferritin (12.2 nm), catalase (10.0 nm), lactate dehydrogenase (8.2 nm) and serum albumin (7.2 nm).[5b] The marker and samples were prepared to contain 8 µg protein and 1x NativePAGE sample buffer (4x stock, Invitrogen) before loading into separate wells in the gel. The gels were run at 150 V with NativePAGE anode and cathode running buffer (Invitrogen) in the Bolt Mini Gel Tank (Novex Life Technologies) until the dye front approached the end of the gel. The gels were stained for 1 hour using InstantBlue (Expedeon) stain and imaged with the Molecular Imager ChemiDoc XRS+ Imaging System and Image Lab 4.0 Software (Bio-Rad). The relative mobility (R_f) was used to estimate the respective molecular weights and rHDL diameters using FIJI software.[7]

1.5. Transmission electron microscopy

For TEM analysis, the method was adapted from Zhang *et al.* 2011[8], the carbon-coated copper grids (Agar Scientific) were glow-discharged before 3 µL of rHDL sample was pipetted onto the grid (protein concentration approximately 0.25 or 1.0 mg/ml). After 2 minutes the excess sample was blotted with filter paper. Followed by x3 wash steps with 35 µL dH_2O drops on parafilm in quick succession, with blotting in between each step. The grid was negatively stained by inverting onto 3 drops of 2 % (w/v) uranyl acetate (UA) on parafilm in quick succession with 2 minutes on the last drop of UA, and the excess stain blotted after each drop. The sample grid was dried under a heat lamp before imaging. The materials and microscope used were

provided at the Astbury Centre for Structural Molecular Biology at the University of Leeds. The sample grid was imaged using a FEI Tecnai T12 microscope with a 120 keV Lab6 electron source and a Gatan US4000/SP 4k x 4k CCD camera, at 68 k magnification. The images were processed using FIJI software.[7]

1.6. Preparation of oriented rHDL

The rHDL nanoparticles in rHDL buffer, pH 7.4, with a total volume of 1 ml were deposited evenly across 24 glass cover slips and dried in a desiccator overnight. The glass cover slips (Paul Marienfeld GmbH & Co. KG, Germany; 8 x 22 mm, thickness No. 0 (0.08-0.12mm)) were cleaned by rinsing in MeOH, EtOH and MilliQ grade water and dried at 37°C for 24 – 48 hours. The samples were humidified at 99 % humidity at 37°C for the respective times as in the main text. The cover slips were stacked and wrapped in cling film immediately before NMR analysis. The human plasma HDL sample (10 mg, Merck) was in solution containing 150 mM NaCl, 0.01 % EDTA, pH 7.4, and contained approximately 5 mg protein. The sample was diluted to 1 ml and prepared as described above.

1.7. PEG Precipitation of rHDL

For ^{13}C CP-MAS NMR analysis the rHDL samples were precipitated using PEG.[9] PEG-6000 (40 % (w/v) stock) was added to the rHDL samples to a final concentration of 24 % (w/v). The samples were incubated at 4°C and inverted to mix for at least 48 hours to ensure the sample precipitated. To pellet the precipitate and remove the supernatant, the samples (0.8 ml) were centrifuged at 41 krpm at 4°C for 2 hours in an SW 55 Ti rotor (Beckman Coulter). The PEG precipitated sample was stored at 4°C until loaded into a 3.2 mm diameter magic-angle spinning rotor.

1.8. Circular dichroism

Samples were analysed using circular dichroism of lipid-free and fractionated rHDL apoA-I WT, apoA-I R173C and apoA-I L144R. The samples were in rHDL buffer, pH 7.4 at a protein concentration of 0.1 mg/ml (3.6 µM) and 300 µL volume. The data was collected on a Chirascan plus qCD spectrometer (Applied Photophysics). The quartz cuvette (Starna scientific) path length was 1 mm, and the wavelength range was 180 – 260 nm, bandwidth 1 nm, time per point 0.5 s, and step size 1 nm. Initially, the samples were compared at 37°C and scanned in triplicate. The background CD signals were collected in triplicate and subtracted from all spectra

before analysis. The lipid-free sample background signal was rHDL buffer pH 7.4, and the rHDL sample background signal was POPC SUVs produced by tip sonication and diluted to the appropriate sample concentration.

To check the UV stability of lipid-free and lipid-bound apoA-I WT, R173C and L144R proteins samples were scanned 50 times in succession. To determine the melting temperature the samples were heated from 25 – 95°C in 1°C increments, at a rate of 1°C/min with a tolerance of 0.1^{o}C, and cooled to 25°C following the method of Giudice *et al*.[10]. The secondary structure estimations (SSE) were determined using CDApps software[11] with the CONTINLL algorithm and the SMP56 (43 soluble + 13 membrane proteins) basis set (190-240 nm).

The melting temperature(s) (T_m) of each sample was determined using Global 3 thermal global analysis software (Applied Photophysics). The Global thermodynamic analysis uses multiple wavelength unfolding sigmoidal curve fitting based upon the Gibbs-Helmholtz equation[12] (Applied Photophysics) and was previously used to analyse apoA-I WT thermal properties.[1]

Additionally, the unfolding curves at 222 nm and the corresponding first derivatives were plotted at each temperature to determine the number of transitions indicated by the number of peaks in the first derivative plot.[12] The T_m(s) can be determined using the peak maxima (T_m) in the first derivative plot which correspond to the T_m at the midpoint of the sigmoidal unfolding curve.[12] The approximate T_m(s) determined from the unfolding curve and first derivatives were similar to each other and the Global analysis T_m(s). Therefore, the transitions were determined to be monophasic or biphasic and the unfolding curves were fitted with the Boltzmann (sigmoidal) and BiDoseResponse (double sigmoidal) fits using OriginPro 2018 software, respectively.

1.9. Dynamic light scattering

Dynamic light scattering (DLS) measurements were used to determine the diameter of rHDL WT and L144R after sodium cholate removal using a Zetasizer Nano ZSP (Malvern) at B23 Diamond Light Source, Oxford, UK. The sample was centrifuged at 2000 x g for 10 minutes to remove MLVs prior to measurement. A 50 µL sample was pipetted into a ZEN0040 micro cuvette cell and the samples were in rHDL buffer, pH

7.0, and for each measurement the protein concentration was 36 µM. Each sample was scanned in triplicate at 25°C at 173° scattering angle. The percentage intensity- and volume-weighted population distributions were used to determine the mean particle size and sample particle distribution, respectively.

The rHDL samples were measured using DLS for the percentage intensity-weighted distribution which was used to determine the mean particle size in a sample. The intensity-weighted distribution was converted to the volume-weighted distribution as larger particles in the former distribution can be represented disproportionately at a high intensity/major particle in the sample, as the intensity percentage scales with the particle size.[13] Therefore, the percentage volume-weighted distribution was used to determine the proportion of the particles at each size in the sample.[13] Minor peaks above 20 nm in the intensity-weighted distribution were absent from the volume-weighted distribution and did not contribute significantly to the particle distribution in the sample. The polydispersity index (PdI) was used to confirm that the samples were suitable for DLS measurement. A PdI > 0.7 indicates a more polydisperse sample and therefore a broad particle size distribution compared to < 0.7 to approaching monodisperse samples (< 0.1).[13]

The measured translational diffusion constant D_t of an ideal spherical particle is inversely proportional to its hydrodynamic radius r_H according to the Stokes-Einstein relation

$$r_H = \frac{k_B T}{6\pi\eta D_t} \qquad [1]$$

where η is the solvent viscosity. Application of this equation to non-ideal systems can underestimate r_H, as is the case for rod-like structures, or overestimate r_H, as is the case for ellipsoidal structures. Perrin modified equation [1] for the case of biaxial ellipsoids of semi-axis radii a and b, such that[14]

$$a = \frac{k_{\boldsymbol{B}} T}{6\pi\eta D_t} S(\rho) \qquad [2]$$

Where $\rho = b/a$ and the shape factor, $S(\rho)$, for an oblate ellipsoid ($b > a$) is given by

$$S(\rho) = (1-\rho^2)^{-\frac{1}{2}} tan^{-1}\left[(1-\rho^2)^{\frac{1}{2}}\right] \qquad [3]$$

According to this modification, the closer the particle is to being spherical, the closer the actual diameter will be to the hydrodynamic diameter calculated from the measured D_t. If the particle deviates from the spherical ideal, i.e., as an oblate ellipsoid (ρ decreases), the hydrodynamic radius calculated from the measured D_t in equation [1] is expected to be overestimated by up to 20 %. The rHDL L144R morphology is approximated by an oblate hemispheroid for which $f = 1 - a/b = 0.5$-0.6. No explicit relationship has, to our knowledge, been developed for such structures, but the surface curvature of these particles is closer to the spherical ideal than is the case for the planar rHDL WT structures. For the case of colloidal discs, a relationship between the disc radius R and an effective average translational diffusion coefficient has been developed,[15]

$$D_t = \frac{1}{3(D_{\parallel} + 2D_{\perp})} = \frac{k_B T}{12\pi\eta R} \qquad [4]$$

Where $D_{\parallel}$ and $D_{\perp}$ are the constants representing diffusion of the discs parallel and perpendicular to the disc normal. Here, DLS measurements of discoidal rHDL L144R particles yielded a mean apparent hydrodynamic diameter, d_H, of 10.5 Å. The diffusion constant of a spherical particle of diameter 6.5 nm is calculated to be 3.77 x 10^{11} m s^{-1} at 298 K and aqueous viscosity of 8.9 x 10^{-4} Pa·s according to equation [1]. If this value is substituted into equation 4, the disc diameter is calculated to be 5.25 Å (i.e., half the diameter calculated using the spherical approximation).

In conclusion, the apparent difference in the size distributions of rHDL WT and rHDL L144R are due, at least in part, to their different, morphologically-dependent diffusion behaviour. This would also explain why no such size difference is apparent from the TEM measurements.

1.10. Solid-state NMR

All measurements were carried out at 25°C on a Bruker Avance 400 spectrometer with an 89 mm bore magnet at a magnetic field strength of 9.3 T. ^{31}P NMR measurements on the oriented rHDL samples were performed with a static, double resonance (H/F and X, tuned to 161 MHz) flat-coil probe (Bruker) with coil

dimensions 9 x 9 x 3 mm. Spectra were acquired after excitation of ^{31}P spins with a 3.5 μs 90° pulse, with 20 kHz continuous wave proton decoupling applied during the acquisition period. Spectra were the result of accumulating between 10,000 and 50,000 transients with a 2 s recycle delay.

The ^{1}H-^{15}N PISEMA spectrum was obtained using the basic pulse sequence described by Wu *et al*.[16] An initial 2.5 μs 90° pulse on ^{1}H was followed by 2 ms ramped cross-polarisation from ^{1}H to ^{15}N at a proton nutation frequency of 40 kHz. Spin exchange at the magic-angle was achieved using frequency-switched Lee-Goldberg cross-polarization at a proton field of 62.5 kHz and matched ^{15}N field of 76 kHz. Proton decoupling at a field of 62.5 kHz was applied during signal acquisition. The spectrum was the result of 32 t_1 increments in the indirect dimension, with averaging of 10,000 transients per increment.

The 2D ^{13}C-^{13}C spectrum of precipitated rHDL was recorded with a HFXY probe operating in double-resonance mode and magic-angle spinning at 8 kHz. Samples were confined in a 3.2 mm zirconium rotor. Hartmann-Hahn cross-polarization was achieved with a 2 ms contact time and 63 kHz proton decoupling with SPINAL-64 was applied during signal acquisition. Spectra were recorded with a 100 ms mixing time during which the proton nutation frequency was adjusted to the MAS frequency of 14 kHz to meet the dipolar-assisted rotational resonance (DARR) condition.[17] Typically, 480 increments were acquired in the indirect (t_1) dimension with 400-600 transients per increment, and the total measurement time varied from 2-7 days depending on the efficiency of rotor packing. Phase-sensitive detection in the indirect dimensions was achieved using the States-TPPI method. Chemical shifts are expressed relative to tetramethylsilane.

1.11. Calculation of ^{31}P NMR lineshapes

1.11.1. Planar and spherical lipid distributions

For a bilayer lipid undergoing rapid rotational diffusion about its long molecular axis, where β is the angle between the principal axis of the rotationally-averaged chemical shift tensor and B_0, the measured ^{31}P NMR frequency is given by

$$\omega = \Delta\delta_{av}(3cos^2\beta - 1)/2 + \delta_{iso} \qquad [5]$$

For POPC in fluid bilayers the isotropic chemical shift δ_{iso} is close to zero and the rotationally averaged full chemical shift anisotropy $\Delta\delta_{av}$ is 45 ppm. The orientationally-dependent NMR frequencies occur at or between the upper limit of 30 ppm and lower limit of -15 ppm. The NMR lineshape is dependent on the relationship between the orientation distribution of the ensemble of lipids undergoing fast molecular motions and the weighted-average frequencies of the molecules in the sample. Formally, the relationship between the spectral density S(ω) and angular distribution P(β) is given by

$$S(\omega)|d\omega| = P(\beta)|d\beta| \quad [6]$$

For a perfectly spherical lipid assembly, such as a liposome, the angular distribution $P(\beta)$ = sin(β) and a spherically-averaged, axially symmetrical powder lineshape is observed. In the case of a fully planar bilayer, β = 0° when the bilayer normal is parallel with B_0 and β = 90° when the bilayer is perpendicular to B_0. In both orientations of the planar bilayer, $P(\beta)$ represents a limited random distribution of lipid orientations about these angles, and narrow Lorentzian lineshapes are observed approximately at the two extreme frequencies, 30 ppm and -15 ppm.

The real part of the NMR signal as a function of time can be calculated according to

$$\mathrm{Re}(S(\mathrm{t})) = \sum_{\beta=0}^{\frac{\pi}{2}} P\,(\beta) cos(\omega t) \quad [7]$$

and the NMR lineshape is obtained by Fourier transformation of the complex signal. Here, all calculated signals included the broadening function exp(-t/T_2).

1.11.2. Lineshapes calculated from MD models

A ^{31}P NMR line shape was simulated based on the atomistic, microsecond MD simulations of HDL comprising 200:20:2 POPC:cholesterol:apoA-I.[18] The POPC molecules undergo anisotropic rotation about their long molecular axes, in addition to local motional fluctuations and translational motion. For the purpose of the simulation it was assumed that rotational diffusion about the angle β is the dominant process that scales the observed ^{31}P CSA according to equation [5]. Hence, by determining the average inclination angle, β_{av}, of each lipid molecule over a timescale greater than the rotational diffusion timescale (i.e., over the final 1 μs of the trajectory), the

dynamically-averaged ^{31}P NMR frequency of each lipid was determined. To do this, the principal axis of inertia, I_z^{HDL}, of the entire rHDL lipid matrix was assumed to be oriented either parallel with B_0 (representing discrete rHDL particles) or perpendicular to B_0 (representing rouleaux). For each lipid molecule per frame of the MD trajectory, we calculated the angle β between the principal axis of inertia of each POPC molecule, I_z^{POPC}, and B_0. This was repeated over 64 frames spanning the final 1 μs of the 10 μs MD trajectory, during which time the RMSD for the protein conformation plateaus at around 6 Å and further small variations in the RMSD value reflect fluctuations in the disc shape. Angle β_{av} calculated over this period was substituted into equation [5] to determine the time-averaged ^{31}P frequency for each of the 200 POPC molecules, giving the line shapes shown in Figure 1C of the main text. We found that this approach is more sensitive to the average orientation of each lipid molecule than an alternative method in which the motional averaging of the full CSA tensor of each lipid molecule was considered.

1.11.3. The hemispheroid approximation

For cases where the surface curvature of the rHDL lipid bilayer was approximated by a hemispheroid (concave or convex), the density of probability was calculated according to (Ref [17] of the main text)

$$P(\beta) = \frac{2\pi b^2 sin\beta}{[sin^2\beta + r^2 cos^2\beta]^2}, \qquad [8]$$

where $r = b/a$ and a and b are defined in Fig 1. It is assumed that the surface curvature is represented by an oblate hemispheroid (i.e., $a < b$). When axis a is parallel with B_0 (i.e., approximating the case of discrete rHDL particles), the NMR line shape is readily calculated from equations [5], [6] and [8] by summation over all angles β from 0° to 90°. When axis a is perpendicular to B_0 (i.e., approximating the case of rouleaux), for each angle β the lipids describe a circular distribution of angles, α, relative to B_0, where α takes values from +(90°-β) to –(90°-β) (Scheme 1). Hence the lineshape is calculated from equations [5] and [6] by substitution of α into equation [8] and by summation over all angles, α and β.

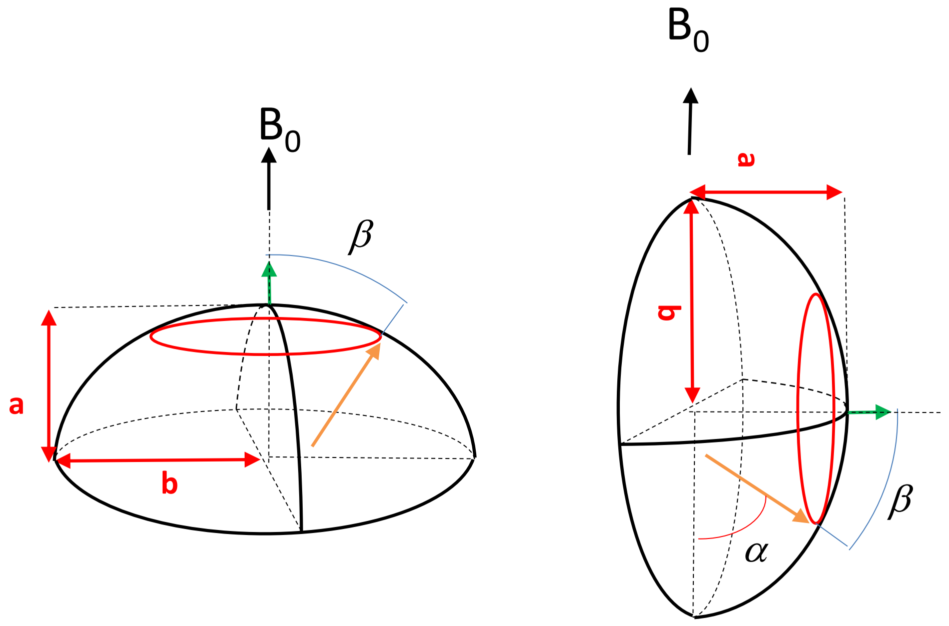

Scheme 1. Surface curvature of rHDL lipid bilayer hemispheroid approximation with axis *a* parallel (left) and perpendicular (right) to B_0, respectively.

1.11.4. Simulations of discoidal particles.

For simulations of lineshapes for rHDL comprising cholesterol, the particle shape was considered to have two components approximating a discoidal morphology. An inner circular core of a planar lipid bilayer of radius *c* is surrounded by outer lipids having orientational distributions described by a partial torus of width *t* (as defined in Figure 4 of the main text). The extent of curvature of the outer lipid molecules is defined by angle $\beta_{t,}$ defining the maximum tilt angle of the lipids away from the HDL principal axis I_z^{HDL}. The condition was set that the curvature of the outer lipids does not alter either the overall diameter *d* (taken to be 11 nm) or thickness *b* (taken to be 5.5 nm) of the particles. In order to maintain this condition, the radius of the inner core must therefore contract as β_t increases, as

$$c = 0.5(d - bsin\beta_t) \qquad [9]$$

The fraction of lipids, f_t, occupying the partial torus is given by

$$f_t = \frac{b(2\beta_t c + bsin\beta_t)}{2c^2 + b(2\beta_t c + bsin\beta_t)} \qquad [10]$$

and the fraction of lipids occupying the planar bilayer core is $f_c = 1 - f_t$.

When I_z^{HDL} is parallel with B_0, the density of probability of lipids in the outer torus component is given as (Ref [17] of the main text)

$$P_t(\beta) = 0.5\pi b(c + 0.5 b sin\beta) \quad [11]$$

where 0 ≤ β ≤ β_t. In this orientation the line shape could be calculated by summation of the angular distributions in the planar bilayer (for which β = 0°) and torus components (for which 0 ≤ β ≤ β_t), using equations [9]-[11]. When I_z^{HDL} is oriented perpendicular to B_0 it was necessary to use a numerical approach in which the shape of the disc was described by unit vectors representing the inner lipid bilayer component and outer torus component. The line shape was calculated from the angles β between the unit vectors and the *z*-axis representing the direction of the applied magnetic field.

1.12 Calculations of two dimensional NMR spectra

Calculated ^1H-^{15}N PISEMA NMR spectra and 2D ^{13}C-^{13}C NMR spectra were based on a single model obtained with atomistic, microsecond timescale MD simulations of HDL comprising 200:20:2 POPC:cholesterol:apoA-I.[18]

For the PISEMA spectrum, the atomic coordinates of HDL in the final frame of the MD simulation were rotated into a coordinate system in which the principal axis I_z^{HDL} was assumed to align with the direction of the applied magnetic field. In this coordinate system, the ^{15}N chemical shift, δ_i, of each apoA-I backbone amide group, *i*, is calculated as

$$\delta_i = \delta_{11} sin^2\Theta cos^2\Phi + \delta_{22} sin^2\Theta sin^2\Phi + \delta_{33} cos^2\Theta, \quad [12]$$

where δ_{11}, δ_{22} and δ_{33} are the main elements of the amide ^{15}N chemical shift tensor (taken to be 60, 75 and 225 ppm, respectively) and defined as (δ_{33} - δ_{iso}) ≥ (δ_{11} - δ_{iso}) ≥ (δ_{22} - δ_{iso}), with δ_{iso} being the isotropic chemical shift derived according to δ_{iso} = (δ_{11}+δ_{22}+δ_{33})/3. In helical proteins, δ_{33} is directed within the plane of the peptide bond at an angle of about 18° from the N-H bond. Angles Θ and Φ are the polar and azimuthal angles defining the orientation of B_0 in the chemical shift tensor frame. The ^1H-^{15}N dipolar coupling constant, D_i, for each residue *i* was calculated from

$$D_i = D_{max}(3cos^2\theta - 1)/2, \quad [13]$$

where θ is the angle between the N-H bond and B_0 and D_{max} is the maximum value of the dipolar coupling (taken to be 11 kHz).

The calculated 2D ^{13}C-^{13}C NMR spectrum was obtained from ^{13}C chemical shifts predicted from the final frame of the MD simulation[18] by submitting the PDB file of the protein coordinates to the SHIFTX2 server[19] to generate ^{13}C chemical shifts predicted from the protein secondary structure. A 2D spectrum was computed from these frequencies by correlating the backbone amide carbon (C') and Cα chemical shifts with the chemical shifts for Cβ, and other side-chain carbons. The simulations are intended simply to show the predicted positions of the cross-peaks and do not take into account the factors such as the dipolar coupling strength or spin-diffusion that influence their intensity. Hence, all cross-peaks and diagonal peaks were assumed have the same intensity. The time domain signal with the appropriate frequencies was generated in two dimensions, with an exponential line broadening function of 25 Hz, and Fourier transformation was performed to obtain the spectrum. The PISEMA and ^{13}C-^{13}C spectra were generated by C programs written specifically for this purpose. Calculations were carried out in the time domain and Fourier transformed in both dimensions to give the spectrum.

2. Tables

Table S1. Secondary structure contents of lipid-free apoA-I and rHDL WT, R173C and L144R estimated using the CONTINLL algorithm and SMP56 basis set, average of 3 scans.

Sample	Secondary structure estimate (%)			
	α-helix	β-strand	turns	unordered
apoA-I WT	51.6	6.1	15.7	26.6
apoA-I R173C	46.2	5.3	17.9	30.6
apoA-I L144R	54.9	6.3	18.3	20.5
rHDL WT	66.6	1.9	11.1	20.3
rHDL R173C	73.8	19.1	7.1	0
rHDL L144R	54.3	3.1	12.1	30.6

Table S2. Size analysis of apoA-I and rHDL-WT particles (1.15 g/mL fraction) by non-denaturing native gel electrophoresis. A linear relationship exists between R_f and log(MW) (R^2 = 0.9848) and between R_f and log(*d*) (R^2 =0.9189).

**Marker**	**R_f**	**MW (kDa)**	**log(MW)**	**Diameter *d* (nm)**	**log(*d*)**
1	0.22	669.00	2.83	17.00	1.23
2	0.33	440.00	2.64	12.20	1.09
3	0.41	232.00	2.37	10.00	1.00
4	0.52	140.00	2.15	8.20	0.91
5	0.70	66.00	1.82	7.10	0.85

	**R_f**	**MW (kDa)**	**log(MW)**	**Diameter *d* (nm)**	**log(*d*)**
**ApoA-I WT**	0.89	**24.52**	1.39	4.63	0.67

**rHDL-WT**	**R_f**			**Diameter *d* (nm)**	**log(*d*)**
Upper limit	0.41			**10.95**	1.04
Lower limit	0.52			**8.98**	0.95

Table S3. Size analysis of apoA-I L144R and rHDL-L144R particles (1.15 g/mL fraction) by non-denaturing native gel electrophoresis. A linear relationship exists between R_f and log(MW) (R^2 = 0.9904) and between R_f and log(*d*) (R^2 =0.9452).

Marker	R_f	MW (kDa)	log(MW)	Diameter *d* (nm)	log(*d*)
1	0.23	669.00	2.83	17.00	1.23
2	0.35	440.00	2.64	12.20	1.09
3	0.42	232.00	2.37	10.00	1.00
4	0.54	140.00	2.15	8.20	0.91
5	0.69	66.00	1.82	7.10	0.85

	R_f	MW (kDa)	log(MW)	Diameter *d* (nm)	log(*d*)
**ApoA-I L144R**	0.92	**25.68**	1.41	**4.24**	0.63

rHDL-L144R	R_f			Diameter *d* (nm)	log(*d*)
Upper limit	0.46			**10.09**	1.00
Lower limit	0.50			**9.38**	0.97

Table S4. Melting temperatures (T_m) of lipid-free and rHDL bearing apoA-I WT, apoA-I R173 and apoA-I L144R ± error (^oC).

Sample	Observed (^oC)		Literature (^oC)	
	T_m^1	T_m^2	T_m^1	T_m^2
apoA-I WT	55.1 ± 0.1	-	55.3 ± 0.5[20]<br>55.9 ± 1.4[21]<br>56.0[1]<br>57.3 ± 0.4[6]<br>59 ± 1.0[22]<br>60.0 ± 2.0[23]<br>60 ± 1.5[24]<br>61 ± 1.0[25]	
apoA-I R173C	51.4 ± 0.1	-	50.9 ± 1.4[21]	
apoA-I L144R	53.2 ± 0.1	-	-	-
rHDL WT	43.4 ± 0.3	82.9 ± 0.4	46.6 ± 3.6[10]	78.8 ± 0.2[10]
rHDL R173C	47.0 ± 0.4	81.0 ± 0.4	55.9 ± 5.0[10]	72.15 ± 0.4[10]
rHDL L144R	44.6 ± 0.2	76.3 ± 0.1	-	-

3. Supplementary figures

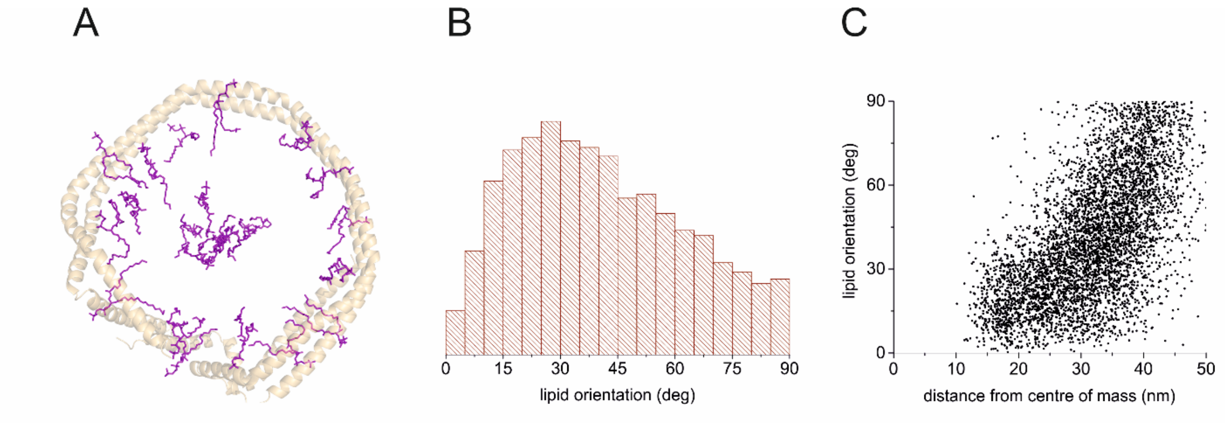

Figure S1. Lipid orientations in molecular dynamics simulations of bilayer assemblies. A: The structural model of 200:20:2 POPC:cholesterol:apoA-I obtained after 10 μs MD simulation (redrawn from Figure 1B), viewed down the principal axis I_z^{HDL} to highlight the orientations of the inner and outer POPC lipids. B: POPC molecular orientations relative to the HDL principal axis measured from the POPC coordinates in the final 1 μs of the MD simulation. C: Relationship between each lipid orientation (defined as the angle of tilt away from I_z^{HDL}) and the distance of its centre of mass from the overall centre of mass of the particle.

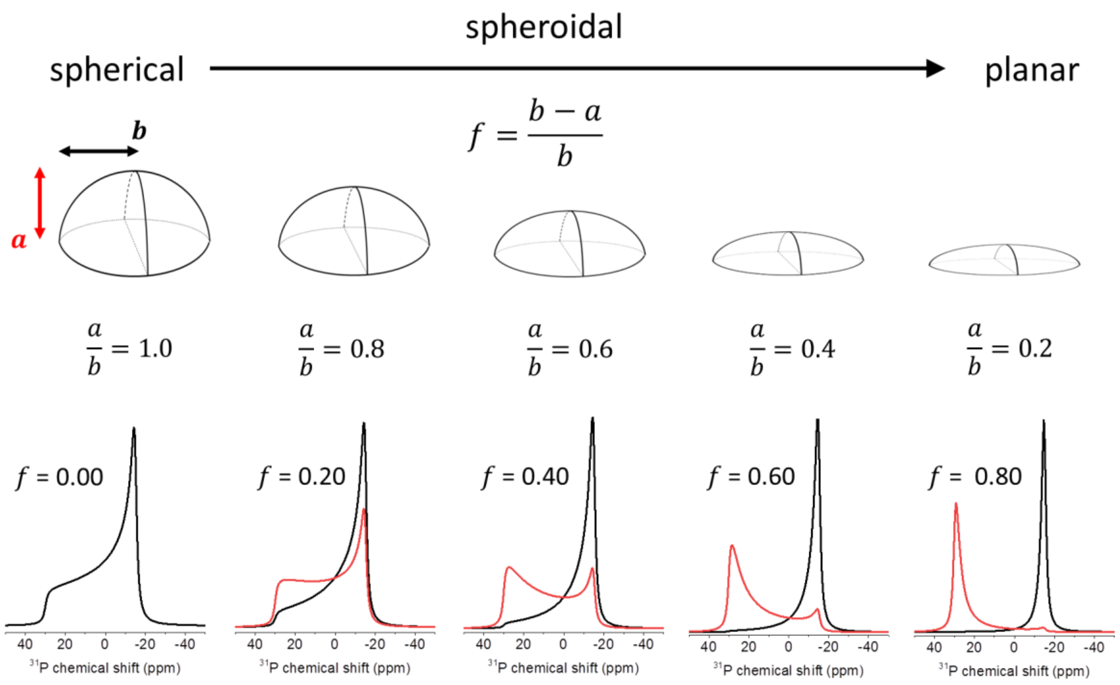

Figure S2. Simulated ^{31}P NMR line shapes (normalised to peak area) for different rHDL phospholipid surface curvatures approximated by an oblate hemispheroid with *a* < *b*. The flattening factor *f*, indicates a planar surface when *f* = 1.0 and a perfect spherical surface when *f* = 0.0. A full ^{31}P spectra width of 45 ppm is assumed for POPC. Red lines correspond to the hemispheroid oriented with axis *a* parallel to B_0, and black lines to axis *a* perpendicular to B_0.

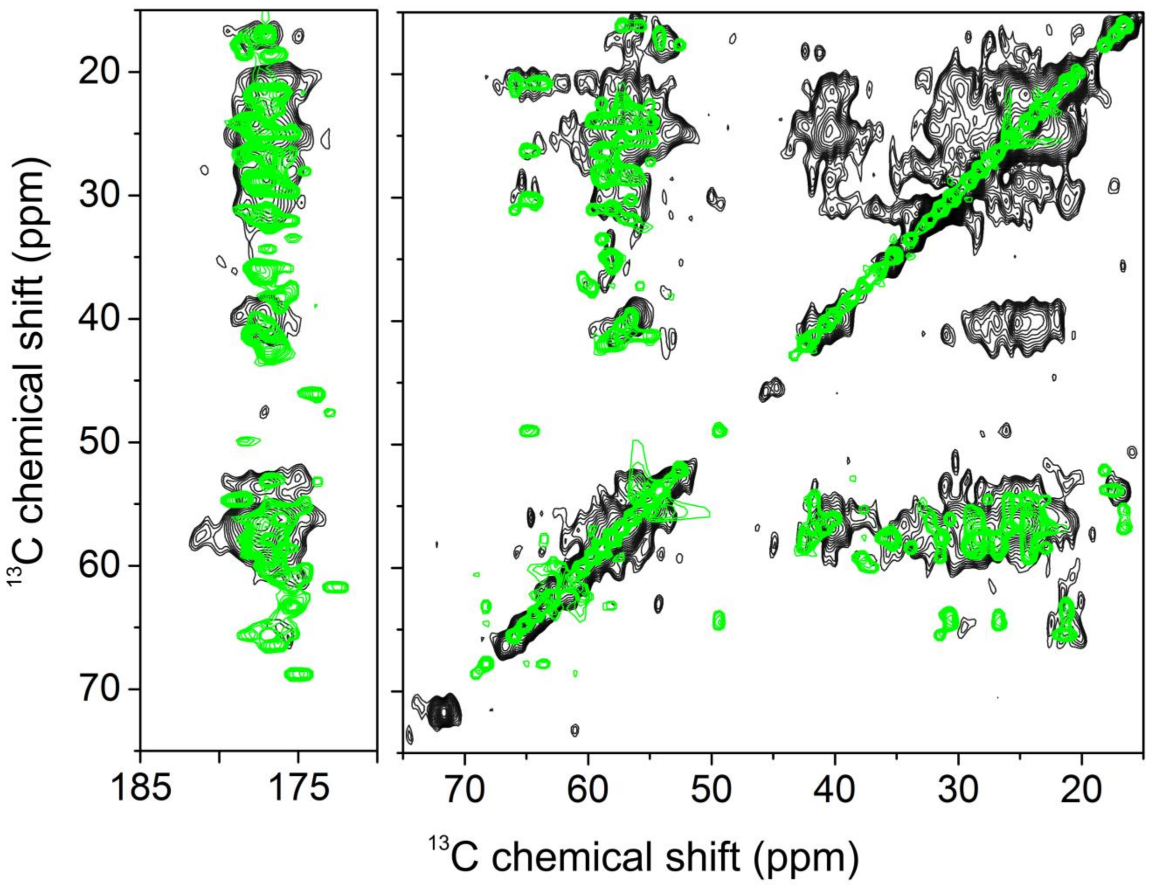

Figure S3. Expanded regions of a ^{13}C-^{13}C dipolar correlation spectrum of PEG-precipitated 200:2 POPC:[U-^{13}C]apoA-I, obtained at a frequency of 100.13 MHz with 100 ms DARR mixing and MAS at 8 kHz. The green contours are simulated and are based on the ^{13}C chemical shifts for apoA-I predicted from the structural model in Figure 1B using the SHIFTX2 server. Cross-peaks correlating Cβ-Cγ, Cγ-Cδ, etc, in the 10-40 ppm region, are not shown because they are relatively insensitive to secondary structure.

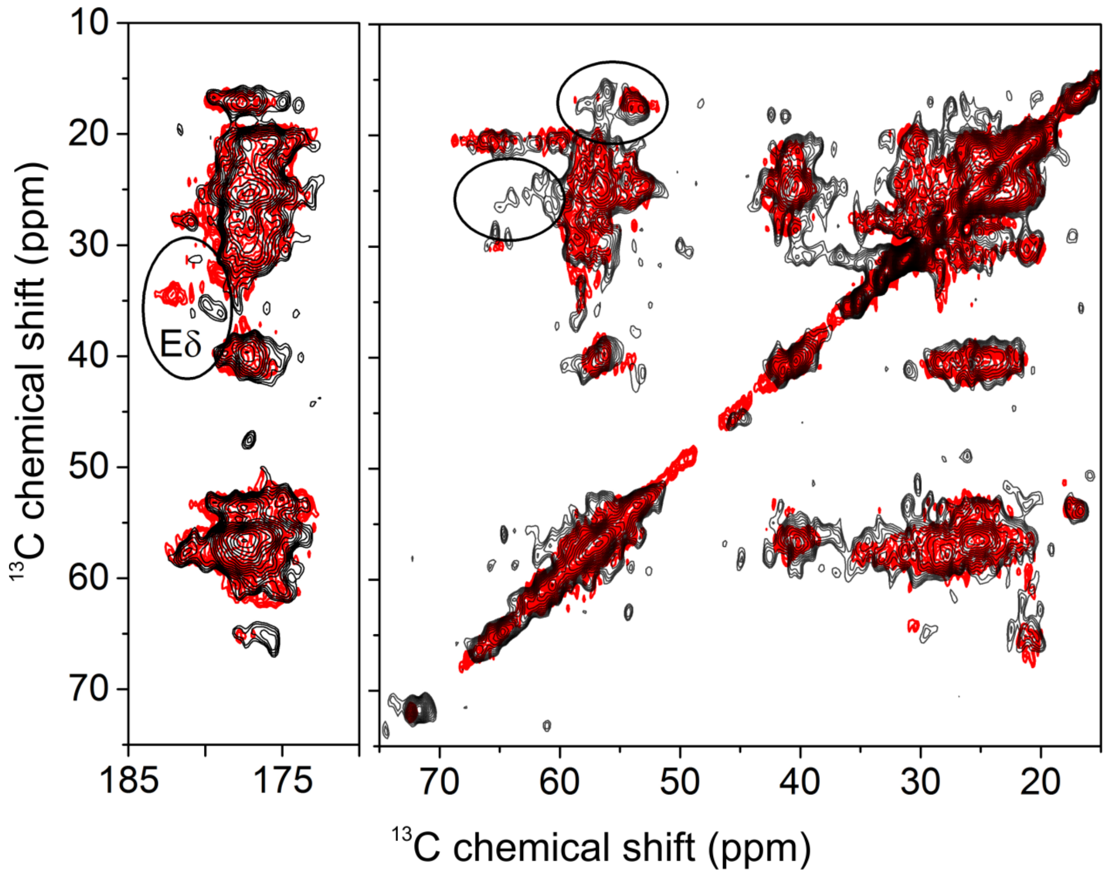

Figure S4. Expanded regions of ^{13}C-^{13}C dipolar correlation spectra of PEG-precipitated 200:2 POPC:[U-^{13}C]apoA-I, obtained at a frequency of 100.13 MHz with 100 ms DARR mixing and MAS at 8 kHz. The black spectrum is from rHDL-WT and the red spectrum is from rHDL-L144R. The circled regions highlight the main differences between the spectra.

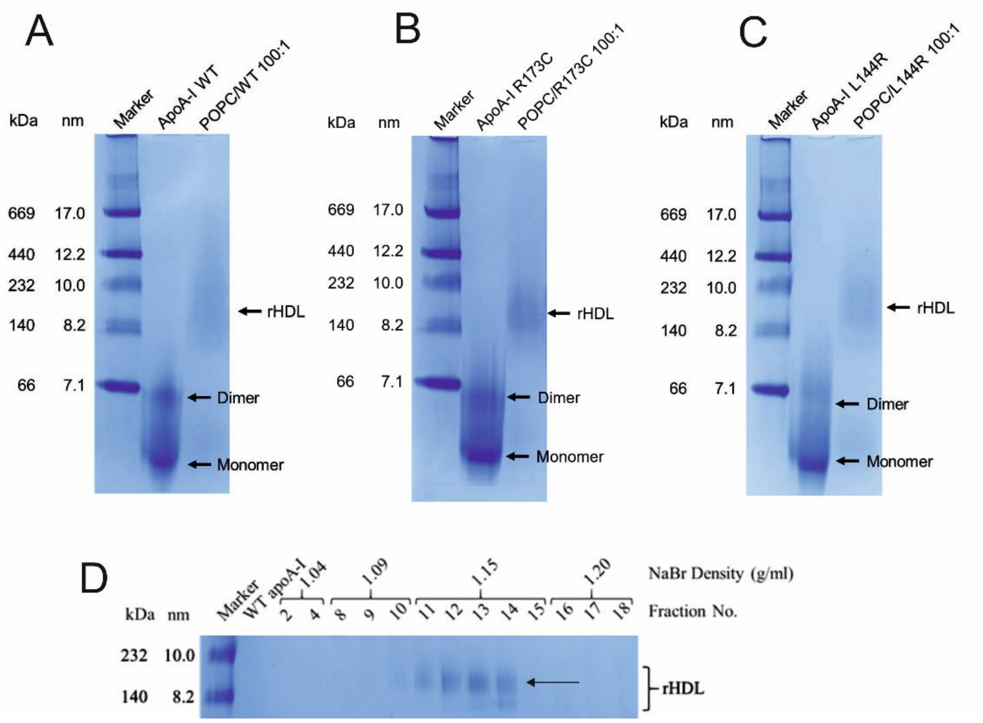

Figure S5. Particle size analysis using non-denaturing gradient gel electrophoresis of rHDL after sodium cholate removal by dialysis. A: rHDL POPC/ apoA-I WT 100:1, B: POPC/apoA-I R173C 100:1, and C: POPC/apoA-I L144R 100:1. D: An example of POPC/WT 100:1 rHDL fractions at 1.04, 1.09, 1.15, 1.20 g/ml NaBr density, separated from lipid-free apoA-I and free lipid using step gradient density ultracentrifugation for PEG precipitation of rHDL. The predominant rHDL species for apoA-I WT, apoA-I R173C and apoA-I L144R have an estimated diameter of 9.1 – 11.3 nm, 9.0 – 10.8, 9.1 – 10.9 nm, respectively. The lipid-free apoA-I WT, apoA-I R173C and L144R indicate the presence of monomer and dimer formation.

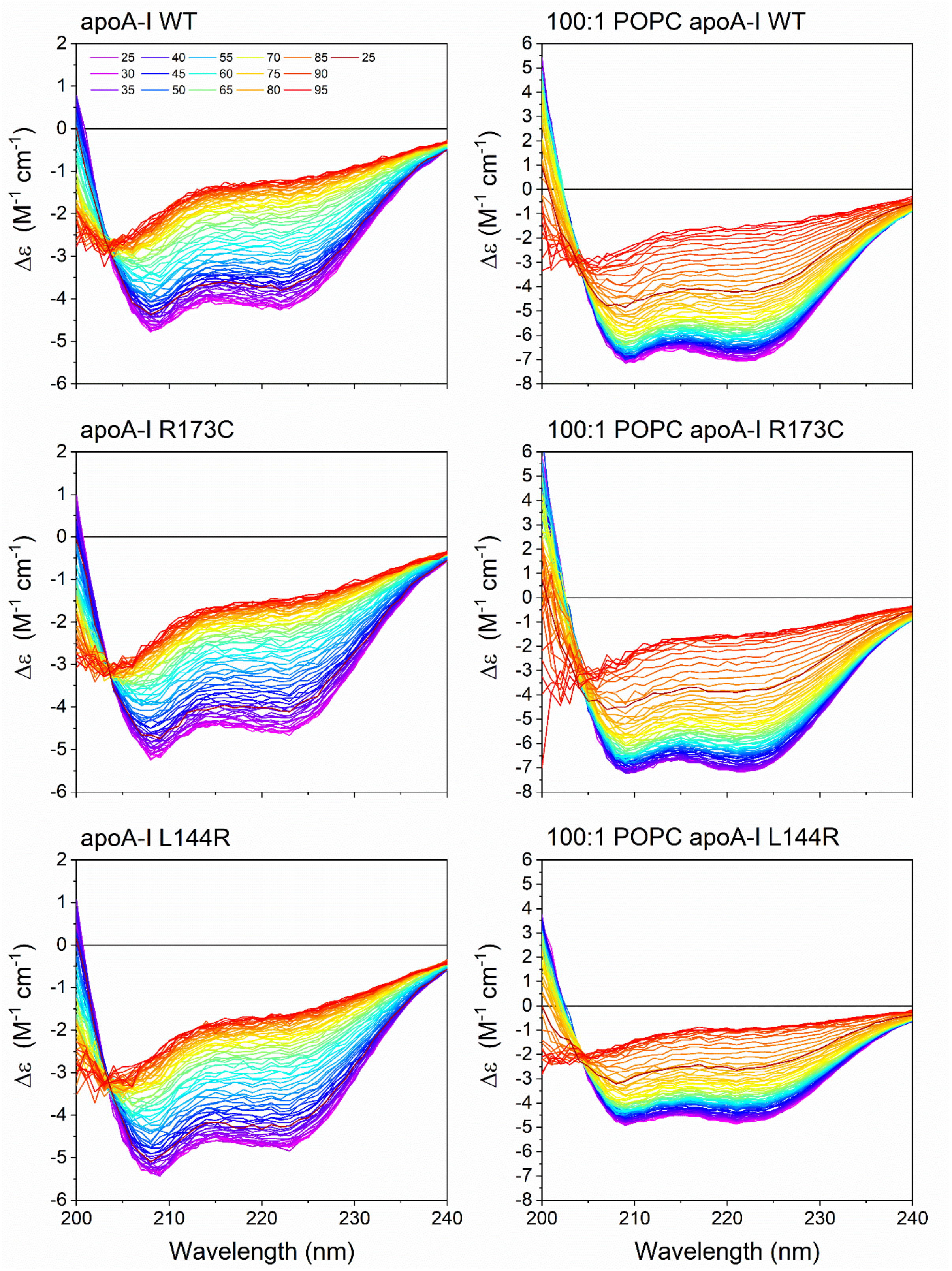

Figure S6. Thermal stability of lipid-free apoA-I mutants and rHDL preparations determined by variable temperature circular dichroism spectroscopy. The spectra were obtained in 1°C increments with the figure legend in 5°C increments and report on structural changes in apoA-I.

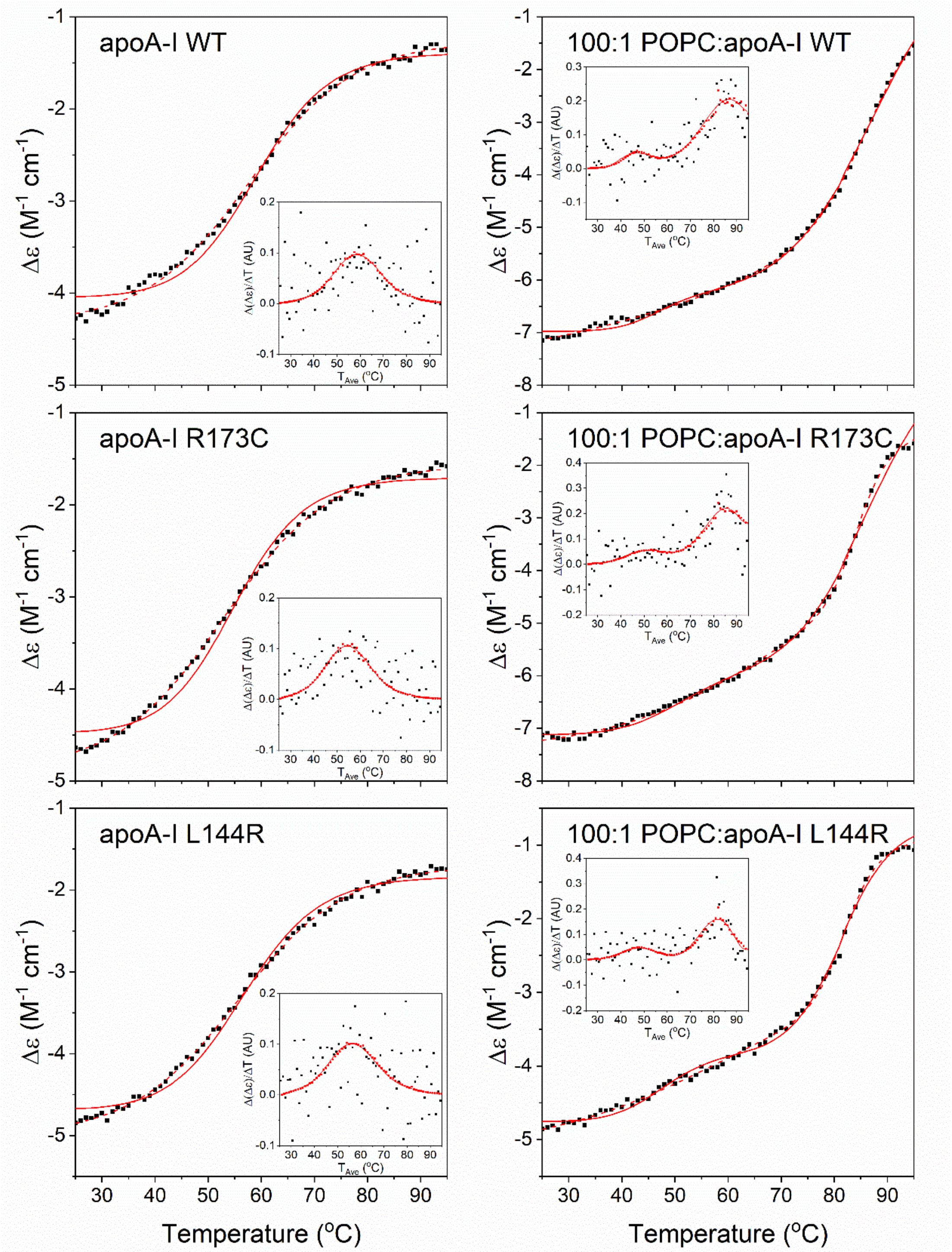

Figure S7. The unfolding curves of the Δε at 222 nm as a function of temperature and the first derivatives (inset) for lipid-free and rHDL 100:1 (M/M) POPC: apoA-I WT, R173C and L144R thermal CD samples. In the unfolding curves: raw data (black) and fit (dashed red line), and Global analysis calculated fit (solid red line). In the first derivative inset (arbitrary units): raw data derivative (black), Global calculated data derivative (red) and polynomial fit (solid red line). The lipid-free samples and rHDL samples show monophasic and biphasic thermal transitions, respectively.

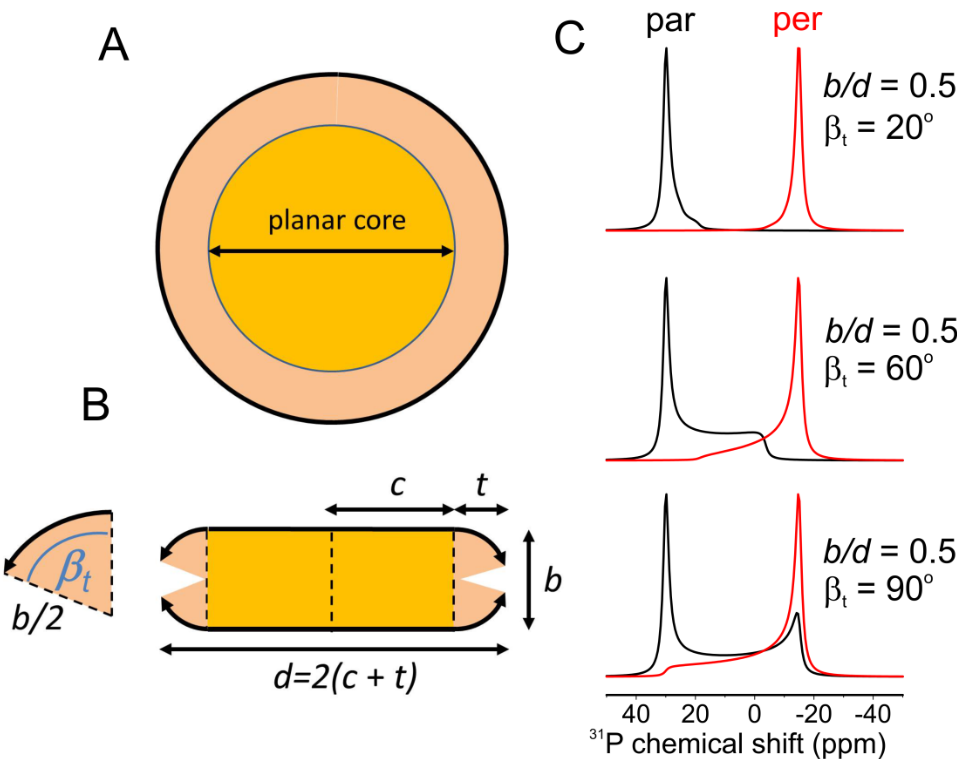

Figure S8. ^{31}P NMR lineshape simulations for an alternative rHDL morphology. A: The core region consists of planar lipid bilayers with a common bilayer normal. The outer lipids tilt away from the bilayer normal and their orientations are given by a spherical distribution of angles β up to a maximum of β_t. As more lipids are recruited into the nanodiscs, β_t increases to a maximum of 90°. At this extreme, the morphology resembles the bicellar structure formed by mixtures of long- and short-chain lipids. If it is assumed that the nanodiscs maintain a constant diameter *d* and thickness *b* as β_m increases, the width of the planar core (2*c*) must decrease accordingly. B: Simulated lineshapes for different values of β_t, for nanodiscs oriented with the principal axis parallel with (black) or perpendicular to (red) B_0. The lineshapes also depend on the ratio *b*/*d* and a constant value of 0.5 was assumed, based on the measured mean diameter (11 nm) and thickness (5.5 nm) from the TEM images.

4. Supplementary References

[1] D. Townsend, E. Hughes, R. Hussain, G. Siligardi, S. Baldock, J. Madine, D. A. Middleton, *Biochemistry* **2017**, *56*, 1632-1644.

[2] J. Chiu, P. E. March, R. Lee, D. Tillett, *Nucleic Acids Res* **2004**, *32*.

[3] I. Sengupta, J. B. Udgaonkar, *Protein Expr Purif* **2017**, *140*, 1-7.

[4] A. Jonas, *Methods in Enzymology* **1986**, *128*, 553-582.

[5] aV. N. Schumaker, D. L. Puppione, *Methods Enzymol.* **1986**, *128*, 155-170; bG. Cavigiolio, B. Shao, E. G. Geier, G. Ren, J. W. Heinecke, M. N. Oda, *Biochemistry* **2008**, *47*, 4770-4779; cJ. L. Kelley, A. W. Kruski, *Methods Enzymol.* **1986**, *128*, 170-181.

[6] J. Dalla-Riva, J. O. Lagerstedt, J. Petrlova, *PLoS One* **2015**, *10*.

[7] J. Schindelin, I. Arganda-Carreras, E. Frise, V. Kaynig, M. Longair, T. Pietzsch, S. Preibisch, C. Rueden, S. Saalfeld, B. Schmid, J. Y. Tinevez, D. J. White, V. Hartenstein, K. Eliceiri, P. Tomancak, A. Cardona, *Nat Methods* **2012**, *9*, 676-682.

[8] L. Zhang, J. Song, G. Cavigiolio, B. Y. Ishida, S. L. Zhang, J. P. Kane, K. H. Weisgraber, M. N. Oda, K. A. Rye, H. J. Pownall, G. Ren, *J. Lipid Res.* **2011**, *52*, 175-184.

[9] K. Mors, C. Roos, F. Scholz, J. Wachtveitl, V. Dotsch, F. Bernhard, C. Glaubitz, *Biochimica Et Biophysica Acta-Biomembranes* **2013**, *1828*, 1222-1229.

[10] R. D. Giudice, O. Nilsson, J. Domingo-Espin, J. O. Lagerstedt, *Sci. Rep.* **2017**, *7*, 13540.

[11] R. Hussain, K. Benning, D. Myatt, T. Javorfi, E. Longo, T. R. Rudd, B. Pulford, G. Siligardi, *J Synchrotron Radiat* **2015**, *22*, 862-862.

[12] N. J. Greenfield, *Nat. Protoc.* **2006**, *1*, 2527-2535.

[13] J. Stetefeld, S. A. McKenna, T. R. Patel, *Biophys Rev* **2016**, *8*, 409-427.

[14] aF. Perrin, *Journal De Physique Et Le Radium* **1934**, *5*, 497-511; bF. Perrin, *Journal De Physique Et Le Radium* **1936**, *7*, 1-11.

[15] S. Jabbari-Farouji, E. Eiser, G. H. Wegdam, D. Bonn, *Journal of Physics-Condensed Matter* **2004**, *16*, L471-L477.

[16] C. H. Wu, A. Ramamoorthy, S. J. Opella, *J. Magn. Reson., Ser A* **1994**, *109*, 270-272.

[17] K. Takegoshi, S. Nakamura, T. Terao, *Chem. Phys. Lett.* **2001**, *344*, 631-637.

[18] M. Pourmousa, H. D. Song, Y. He, J. W. Heinecke, J. P. Segrest, R. W. Pastor, *Proc. Natl. Acad. Sci. U. S. A.* **2018**, *115*, 5163-5168.

[19] B. Han, Y. F. Liu, S. W. Ginzinger, D. S. Wishart, *J. Biomol. NMR* **2011**, *50*, 43-57.

[20] R. Del Giudice, A. Arciello, F. Itri, A. Merlino, M. Monti, M. Buonanno, A. Penco, D. Canetti, G. Petruk, S. M. Monti, A. Relini, P. Pucci, R. Piccoli, D. M. Monti, *Biochim Biophys Acta* **2016**, *1860*, 434-444.

[21] J. Petrlova, J. Dalla-Riva, M. Morgelin, M. Lindahl, E. Krupinska, K. G. Stenkula, J. C. Voss, J. O. Lagerstedt, *PLoS One* **2014**, *9*, e96150.

[22] Y. Fang, O. Gursky, D. Atkinson, *Biochemistry* **2003**, *42*, 13260-13268.

[23] Y. Fang, O. Gursky, D. Atkinson, *Biochemistry* **2003**, *42*, 6881-6890.

[24] H. Saito, P. Dhanasekaran, D. Nguyen, P. Holvoet, S. Lund-Katz, M. C. Phillips, *J Biol Chem* **2003**, *278*, 23227-23232.

[25] X. H. Mei, D. Atkinson, *Journal of Biological Chemistry* **2011**, *286*, 38570-38582.
